# Linking Eight Decades of Canadian Census Collections.

**DOI:** 10.23889/ijpds.v7i3.2076

**Published:** 2022-08-25

**Authors:** Jeremy Foxcroft, Kris Inwood, Luiza Antonie

**Affiliations:** 1 University of Guelph

## Introduction

Linking the many decades of census data collected during Canada’s settlement allows researchers to investigate the movement patterns of early settlers, changes in regional demographics, and intergenerational mobility. This research leverages new methodologies and previously untranscribed individual attributes to link for the first time Canadian censuses from 1852 to 1921.

## Approach

This work aims to build upon prior efforts (1871-1901 linking) to link the decennial Canadian censuses spanning from 1852 to 1921. We use a more complete transcription of the censuses, that has recently become available to researchers through The Canadian People’s project.

Our approach to this task begins with reproducing the results of previous work using data from this new transcription. From there, we add additional time-invariant individual characteristics as features to our classification model. We also explore newer methodologies designed to leverage household information during the linkage process, with the goal of increasing the linkage rate.

## Results

We describe the differences between the different methodologies we use, and the steps we took to clean and standardize the data. We compare the links produced by the different methodologies in terms of the number of links yielded, their quality (false positive rate), and certain aspects of the bias present in the resulting collections of links. We discuss the challenges and potential approaches to dealing with sections of the population who remain difficult to link.

We expect the advancements in record linkage methodologies for historical populations coupled with this more complete transcription of the censuses to offer advantages over prior approaches when linking these censuses. We expect the resulting linked data to offer new insight into Canada during this time period.

## Conclusions/Implications

The resulting collection of linked data over this time period should characterize approximately three generations of early Canadians. This linked data will be passed on to other researchers and will allow us to better understand the changing experiences of the Canadian population during these early stages of Canada’s development.

